# Cell origin and niche availability dictate the capacity of peritoneal macrophages to colonize the cavity and omentum

**DOI:** 10.1111/imm.13483

**Published:** 2022-05-10

**Authors:** Pieter A. Louwe, Stuart J. Forbes, Cécile Bénézech, Clare Pridans, Stephen J. Jenkins

**Affiliations:** 1 Queens Medical Research Institute, University of Edinburgh Centre for Inflammation Research, Edinburgh, UK; 2 Laboratory of Myeloid Cell Biology in Tissue Damage and Inflammation, VIB Center for Inflammation Research, Ghent, Belgium; 3 Centre for Regenerative Medicine, University of Edinburgh, Edinburgh, UK; 4 Queens Medical Research Institute, University of Edinburgh Centre for Cardiovascular Science, Edinburgh, UK; 5 Simons Initiative for the Developing Brain, Centre for Discovery Brain Sciences, University of Edinburgh, Edinburgh, UK

**Keywords:** immune homeostasis, inflammation, macrophage, omentum, peritoneal, trafficking

## Abstract

The relationship between macrophages of the peritoneal cavity and the adjacent omentum remains poorly understood. Here, we describe two populations of omental macrophages distinguished by CD102 expression and use an adoptive cell transfer approach to investigate whether these arise from peritoneal macrophages, and whether this depends upon inflammatory status, the origin of peritoneal macrophages and availability of the omental niches. We show that whereas established resident peritoneal macrophages largely fail to migrate to the omentum, monocyte-derived resident cells readily migrate and form a substantial component of omental CD102^+^ macrophages in the months following resolution of peritoneal inflammation. In contrast, both populations had the capacity to migrate to the omentum in the absence of endogenous peritoneal and omental macrophages. However, inflammatory macrophages expanded more effectively and more efficiently repopulated both CD102^+^ and CD102^−^ omental populations, whereas established resident macrophages partially reconstituted the omental niche via recruitment of monocytes. Hence, cell origin determines the migration of peritoneal macrophages to the omentum and predisposes established resident macrophages to drive infiltration of monocyte-derived cells.

## Introduction

The peritoneal cavity is the fluid-filled space within the peritoneum, a continuous mesothelial membrane that covers the wall and organs of the abdomen. The cavity contains a complex mixture of immune cells that, together with an adipose immune tissue layer called the omentum, provide protection against infection and maintenance of cavity homeostasis [1–3]. Approximately a third of cells within peritoneal fluid are phagocytes. In mice, these are dominated by a population of F4/80^hi^CD102^+^ resident macrophages, which are maintained by longevity, proliferation and replenishment by monocytes [1,4,5]. Much of the transcriptional and functional behaviour of cavity macrophages is driven by retinoid X receptors and the transcription factor GATA6 in response to local production of the vitamin A metabolite retinoic acid [6–9]. Despite playing key roles in many peritoneal diseases [1,10–13] our understanding of the cellular interactions that regulate the identity and maintenance of these macrophages remains incomplete.

Peritoneal macrophages move freely under constant flow [12,14]. Hence, most signals that program the identity of these cells are considered present in peritoneal fluid. Consistent with this hypothesis, conditioning with retinoic acid and supernatant from cultured omenta drives expression of numerous peritoneal macrophage-specific genes in vitro [6,8]. However, evidence suggests cell-to-cell inter-actions with surrounding mesothelium or even migration through immune cell clusters within the omentum, termed fat-associated lymphoid clusters (FALCs), may also facilitate peritoneal macrophage survival and identity [6,8]. For example, *in vitro* expression of certain peritoneal macrophage identity genes requires direct interaction between peritoneal macrophages and omental/mesothelial cells [8]. Notably, monocytes deficient in *Gata6* fail to colonize the cavity but simultaneously accumulate in the omental FALCs, which has been taken as evidence that monocytes mature and up-regulate GATA6 in the omentum prior to emigration into the cavity [6]. Consistent with this, the omentum is inhabited by a population of macrophages that resemble those in the cavity, including sharing expression of CD102, F4/80, GATA6 and the resident marker TIM4 [6,15,16] but which are similarly or more rapidly replenished from the bone marrow (BM) [5]. These data seemingly support the historical perspective of the omentum as the site of generation of peritoneal macrophages [3]. Conversely, resident peritoneal macrophages rapidly migrate to the omentum during the acute phase of peritoneal inflammation [2,6,17]. Hence, while the omentum may be a site of generation and programming of peritoneal macrophages during health, a migratory route from the cavity to omentum also exists for these cells during inflammation.

These findings raise many questions, particularly whether maintenance of established resident peritoneal macrophages requires migration between these sites, or whether resident omental macrophages may actually arise in the cavity. The importance of cell origin in these processes is also unclear. Notably, resident F4/80^hi^CD102^+^ peritoneal macrophages comprise multiple transcriptionally distinct subsets whose identity is related to their recency of monocyte origin [4,5]. Moreover, we and others have demonstrated that monocyte-derived inflammatory macrophages recruited during peritoneal inflammation persist alongside established resident macrophages for many months thereafter [18,19], including following abdominal surgery [5]. Although these recruited cells gradually adopt a resident GATA6^+^F4/80^hi^CD102^+^ phenotype, conversion is slow and incomplete, and for many months they exhibit an altered transcriptional and functional signature akin to monocyte-derived resident macrophages recruited under non-inflammatory conditions, including expressing less GATA6 and GATA6-regulated genes and proliferating more [19]. Whether these differences are functionally important for tissue homeostasis is unclear. Hence, determining migratory routes between the cavity and omentum following inflammation may help us understand what regulates the long-term fate of inflammatory macrophages in the peritoneal cavity and reveal processes involved in the maintenance of peritoneal and omental macrophages in general.

Here, we utilized an adoptive transfer method to definitively track the migration of established and inflammation-elicited resident peritoneal macrophages to the omentum. We found that inflammation-elicited but not established resident macrophages gradually migrate to the omentum under physiological conditions and assume the phenotype CD102^+^ omental macrophages. Inflammation-elicited macrophages also readily expand and efficiently repopulate multiple omental macrophage populations in mice lacking endogenous macrophages. In contrast, established resident macrophages expand less well in the cavity and consequently migrate less readily to the omentum and instead partially reconstitute the omentum by recruiting immature macrophages/monocytes. Hence, our data highlight striking functional differences between established and monocyte-derived resident peritoneal macrophages and suggest the omentum acts as shared niche for monocyte-derived peritoneal macrophages.

## Materials and Methods

### Animal experiments

C57BL/6JCrl, congenic CD45.1^+^CD45.2^+^ (B6.SJL-PtprcaPep3b/BoyJ x C57BL/6JCrl), *Csf1r*^ΔFIRE/ΔFIRE^, and *Csf1r*^+/+^ and *Csf1r*^+/ΔFIRE^ littermate mice [20], and *Ccr2*^−/−^ mice were bred and maintained in specific pathogen-free facilities at the University of Edinburgh, UK. Sex and age-matched mice were used in all experiments. Animal experiments were performed under licence by the UK Home Office and received ethical approval from the University of Edinburgh Animal Welfare and Ethical Review Body. All animal experiments were performed in accordance with the ethical regulations for animal testing and research as set out by the UK Animals (Scientific Procedures) Act of 1986.

### Generation of tissue-protected BM chimeric mice

CD45.1^+^CD45.2^+^ mice were anaesthetized and exposed to 9.5Gy g-irradiation with all but the hind legs or the head and thorax protected by a 5 cm lead shield. The following day animals were reconstituted with 2–5 x 10^6^ BM cells from WT C57BL/6JCrl mice, *Ccr2*^−/−^ mice or *Ccr2*^+/+^ littermate controls. Chimerism was assessed approximately 8 weeks after reconstitution. For wild-type chimeras, chimerism of tissue cells was expressed as relative to chimerism of Ly6C^+^ blood monocytes, whereas actual frequencies of donor cells was provided for experiments using *Ccr2*^−/−^ BM.

### Sterile peritoneal inflammation

To induce mild peritoneal inflammation mice were injected IP with 10 μg of zymosan A (Sigma-Aldrich) suspended in 200 μl Dulbecco’s PBS (dPBS; Invitrogen). Donor mice were injected IP with 250 μl of 700 nM PKH26-PCL suspended in Diluent B (Sigma) 24 h prior to zymosan treatment, and cells harvested for purification by flow-assisted cells sorting (FACS) and transfer on day 3. Recipient mice were either naïve or pre-treated with 10 μg of zymosan A 3 days prior to cell transfer. In some experiments, recipient wild type mice were administered IP with 0.0625 mg Clodronate liposomes (Liposoma) suspended in 250 μl dPBS 8 days prior to receiving IP transfer of FACS-purified donor cells.

### Cell isolation and flow cytometry

Mice were sacrificed by exposure to rising levels of CO_2_. The peritoneal cavity was lavaged with a total of 9 ml icecold wash solution (dPBS containing 2 mM EDTA [Invitrogen] and 1 mM HEPES [Fischer Scientific]). The omentum was then excised, macerated using scissors and digested in 0.5 ml prewarmed enzyme mix (RPMI 1640 with 1% FCS [Gibco] and 1 mg/ml Collagenase D [Roche]) for 35 min on an orbital shaker (37°C). Samples were briefly agitated with a pipette midway through digestion. Following digestion, 2.5 μl of 0.5 M EDTA was added followed by 0.5 ml ice-cold FACS buffer (2 mM EDTA/0.5% BSA in PBS). Samples were then passed through a 100 μm strainer (VWR) and then enumerated using a Casey TT counter (Scharfe). In some experiments, blood was taken from the inferior vena cava following peritoneal lavage or from the tail vein prior to necropsy and immediately mixed in a 10:1 ratio with 0.5 M EDTA before lysis of red blood cells using RBC lysis buffer (Biolegend). For staining, cells were incubated at room temperature for 10 min with zombie aqua viability dye (BioLegend), followed by 10 min on ice with blocking buffer (FACS buffer containing 10% mouse serum with 0.25 μg/ml anti-CD16/CD32 [BioLegend]), and 30 min on ice with a combination of antibodies (Table S1). Cells were then washed with FACS buffer and stained with fluorochrome-conjugated streptavidin (Biolegend). In some experiments, samples were incubated with 5 μl 7AAD Viability Staining Solution (Biolegend) to identify dead cells or fixed using the Foxp3 staining buffer (eBioscience) according to the manufacturer’s protocol. Samples were acquired using FACS LSRFortessa (BD) and analysed using Flowjo (Version 10.4.1, Treestar). For analysis, doublets and dead cells were excluded using Forward scatter area versus height and ZombieAqua or 7AAD, respectively, and T cells, B cells, eosinophils and neutrophils excluded using a Lineage gate (CD3, CD19, Siglec-F, and Ly6G). For analysis of blood from BM chimeric mice, classical monocytes were identified as Ly6C^+^CD115^+^CD11b^+^ following exclusion of Lineage^+^ cells. For FACS, DAPI was used to exclude dead cells. Cells were sorted using a FACSFusion or FACSAria sorting system with a 100 μm sort nozzle.

### Adoptive transfer

For adoptive transfer, cells were stained for cell sorting as described above but under sterile conditions. After FACS purification, cells were counted and 1–2 × 10^5^ cells transferred IP into recipients by injection in 200 μl of dPBS. Where specified, the frequency of donor cells within omental macrophages was also expressed relative to their frequency within F4/80^hi^ cavity macrophages from the same mouse and is referred to as normalized chimerism.

### Macropinocytosis and CSF1R expression

AlexaFluor647 (AF647)-labelled CSF1 (CSF1^AF647^) was prepared as previously described [21]. Briefly, porcine CSF1 [22] was conjugated to AF647 using the AF647 Microscale Protein labeling kit (ThermoFisher Scientific) and sodium azide removed using 7 k MWCO Pierce™ polyacrylamide spin desalting columns (ThermoFisher Scientific). Preservative-free sterile anti-CSF1R mAb (clone AFS98) was purchased from BioServ. To determine CSF1R expression by omental macrophages, mice were injected IP with or without 0.5 mg AFS98 followed by 0.5 μg CSF1^AF647^ or PBS vehicle 2 min later, and then culled 10 min later. For assessment of macropinocytic activity, mice were injected IP with 1 μg Ovalbumin conjugated to Texas Red (ThermoFischer Scientific) in 100 μl PBS or PBS alone and culled after 10 min, as published [21].

### Confocal analysis

Omenta were fixed in 2% neutral buffered formalin for 1 h on ice, before staining in the fridge overnight with antibodies to GATA6 (Cell Signalling; clone D61E4; purified) and F4/80 (Serotec; clone CL:A3-1; purified) or TIM4 (Biolegend; clone RMT4-54; conjugated to AF647) in PBS containing 0.5% BSA, 0.5% triton X-100 (Sigma). Omenta were subsequently stained for 1 h at room temperature with indicated antibodies (Table S2) in PBS containing 0.5% BSA, 0.5% triton X-100, with DAPI added for the final 10 min. Samples were mounted with Fluoromount G (ThermoFischer) and confocal images acquired using a Leica SP8 laser scanning confocal microscope using Leica LAS X software. 3D reconstruction was created using LAS-X-3D (Leica) v3.5.7.23225. *Gata6* expression in CD45^−^ omental cells was determined by analysis of a previously pubished dataset available under accession number GSM4053741 [23].

### Data presentation and statistics

Statistics were performed using Prism 9 (GraphPad Software). Statistical significance was determined using oneway ANOVA followed by Dunnet’s multiple comparisons test, two-way ANOVA followed by Sidak multiple comparisons test, or unpaired Student’s *t*-test. For experiments comparing groups containing fewer than 4 samples, data were log transformed prior to statistical analysis.

## Results

### Omental and peritoneal macrophages are phenotypically distinct

We and others have previously identified a population of CD102^+^ and GATA6^+^ macrophages in murine omentum akin to those within the peritoneal cavity [5,6,16]. Omental macrophages have also been defined based on coexpression of the macrophage markers CD64 and F4/80 [15,16], which encompass both the CD102^+^ macrophages and a population of shorter-lived CD102^−^ macrophages [5]. Peritoneal macrophages are similarly diverse, with a population of short-lived CD102^−^ macrophages that are F4/80^lo^ and MHCII^+^ present alongside the CD102^+^F4/80^hi^ and largely MHCII^−^ resident population [4,24,25]. Notably, intensity of F4/80 and MHCII expression distinguishes macrophages present in the omentum during steady-state from resident peritoneal macrophages that migrate to omentum immediately following inflammation [17,26] suggesting resident peritoneal and omental macrophages are phenotypically distinct.

**Figure 1:**
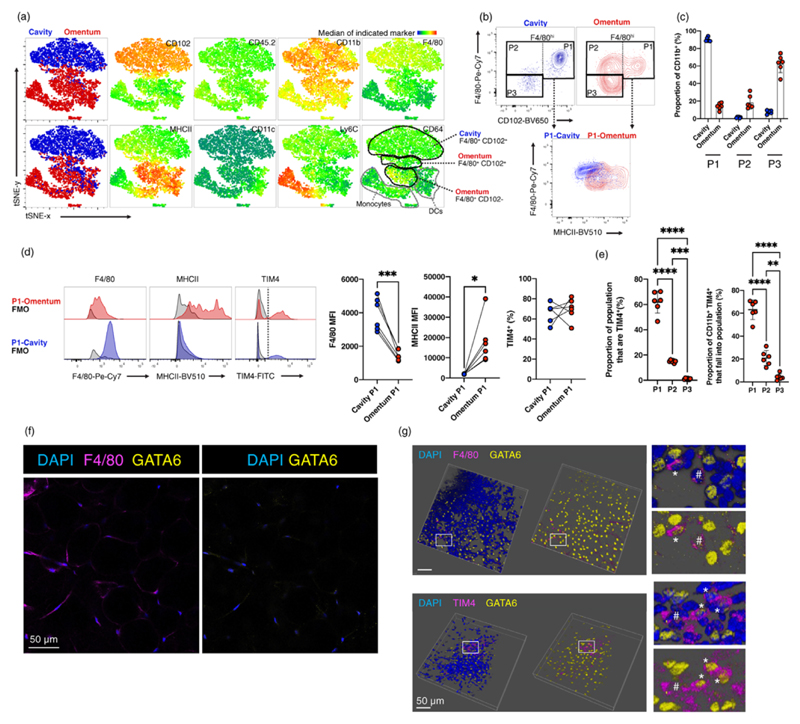
Comparative phenotype of steady-state peritoneal and omental macrophages. (a) tSNE map of three paired concatenated omentum-peritoneal fcs files from naïve mice with an overlay of cavity (blue) and omentum (red) CD11b^+^Lineage^−^ myeloid cells and heatmap for key markers. (b) Representative gating strategy to identify P1 cells in the cavity (blue) and omentum (red). (c) Quantification of data shown in (b) (*n* = 6). (d) Representative expression (left) and quantification (right) of F4/80, MHCII and TIM4 on peritoneal and omental P1 cells (*n* = 6). Statistical significance was determined using paired student’s t test. (e) Proportion of each omental macrophage subset that expresses TIM4 (left) and the proportion of CD11b^+^TIM4^+^ omental myeloid cells that fall into each of the defined subsets (right) (*n* = 6). Statistical significance determined using one-way ANOVA with Tukey’s multiple comparisons test. (f) Representative confocal imaging of omental adipose tissue. (g) Representative confocal imaging and 3D reconstruction of omental FALC, with areas of interest (white box) shown on right. Flow-cytometric data is presented as mean ± standard deviation with each symbol representing individual animals, and was pooled from at least two independent experiments. *p* values are reported as *p < 0.05, **p < 0.01, ***p < 0.001, ****p < 0.0001

Hence, to investigate the relationship between peritoneal and omental macrophages, we first performed a comparative phenotypic assessment of these cells under steady-state conditions. Using CD11b expression and lineage markers to define non-granulocytic myeloid cells in both sites (Figure S1a), subsequent tSNE analysis of CD45, CD11b, CD102, F4/80, CD64, MHCII, Ly6C, and CD11c expression and SSC/FSC characteristics revealed omental and peritoneal CD11b^+^ cells to largely cluster discretely (Figure 1a). Such divergence was evident between the majority of peritoneal and omental F4/80^+^CD102^+^ macrophages but also within the CD102^–^MHCII^hi^ cells, which in the omentum contained a relatively unique population of F4/80^+^CD64^hi^ CD102^−^ macrophages (Figure 1a) that represent those described previously [5,16]. The omentum also contained a predominance of F4/80^−^ cells comprised largely of Ly6C^+^ cells and MHCII^+^ CD11c^+^ cells, likely reflecting omental F4/80^−^CD64^−^CCR2^+^ monocytes and CD11b^+^ conventional dendritic cells (cDC) described previously [15,27].

Based on these findings, we designed a conventional gating strategy to divide both peritoneal cavity and omental CD11b^+^ myeloid cells on the basis of surface expression of CD102 and F4/80, with a discernible population of F4/80^hi^CD102^+^ (P1) and F4/80^lo^ CD102^−^ (P3) cells present in both tissue sites and a population of F4/80^hi^CD102^−^ macrophages (P2) largely restricted to the omentum (Figure 1b,c). Notably, omental P1 cells expressed more MHCII and less F4/80 than their cavity counterparts (Figure 1d) such that these populations appeared distinct based on combined expression of these markers (Figure 1b,d). As reported [15,16], high expression of the macrophage marker CD64 coincided with F4/80 in the omentum (Figure 1a) such that CD64 and F4/80 could be used interchangeably to identify CD102^+^ P1 and CD102^−^ P2 omental macrophages (Figure S1b,c). P3 cells were comprised almost entirely of Ly6C^+^ monocytes and MHCII^+^ cells that were predominantly CD11c^+^ presumed cDC (Figure S1c).

To confirm the macrophage, monocyte and cDC identities of these populations, we injected AF647-labelled porcine CSF1 (Figure S1d) and Texas Red-labelled ovalbumin intraperitoneally (Figure S1f) to measure expression of CSF1R and micropinocytosis, respectively [21]. These methods confirmed that CD102^+^ (P1) and CD102^−^ (P2) populations of omental myeloid cells bound CSF1^AF647^ (Figure S1e) and were highly macropinocytic (Figure S1g), consistent with macrophages, while omental Ly6C^+^ P3 cells were not macropinocytic but bound low levels of CSF1^AF647^ (Figure S1e,g), consistent with monocytes. Combined injection of CSF1^AF647^ and anti-CSF1R antibody confirmed uptake by macrophages and monocytes was at least partly dependent on CSF1 receptor (CSF1R). In contrast, omental Ly6C^−^MHCII^+^ P3 were poorly macropinocytic and bound little CSF1^AF647^ in a receptor dependent manner (Figure S1e,g), consistent with cDCs. Using this new gating strategy, we reanalysed our published dataset of omental cells from tissue-protected BM chimeric mice generated from CD45.1^+^CD45.2^+^ animals given head and thorax-restricted irradiation and CD45.2^+^ donor BM [5], to determine the comparative rates of replenishment of these populations from the BM. Consistent with short-lived monocytes and cDC, omental P3 Ly6C^+^ and MHCII^+^ cells were almost completely replenished from the BM within 8 weeks post-irradiation whereas F4/80^+^CD102^−^ P2 and F4/80^+^CD102^+^ P1 macrophages exhibited progressively less replenishment (Figure S1h). Subsequent analysis in tissue-protected BM chimeric animals given BM from *Ccr2*^−/−^ CD45.2^+^ mice (Figure S1i) confirmed that replenishment of omental Ly6C^+^ monocytes and F4/80^+^ macrophages was wholly dependent on CCR2 expression indicating a monocytic precursor, whereas replenishment of omental DC was only partly dependent on CCR2, and less so than blood monocytes, indicating a non-monocytic origin (Figure S1i). Therefore, using CD102 and F4/80 or CD64, we can delineate two populations of omental macrophages and distinguish these from omental dendritic cells and monocytes.

A distinct approach of subdividing F4/80^+^CD64^+^ omental macrophages into CD163^+^TIM4^+^ and CD163^–^TIM4^–^ cells was recently taken based on single cell transcriptomics of omental cells from mice with metastatic ovarian cancer [15]. Both subsets were also identified in omenta of naïve mice wherein the TIM4^–^CD163^–^ subset exhibited greater replenishment from the BM [15], akin to the CD102^−^ population in our study (Figure S1h). To marry our data with these findings, we compared TIM4 expression by CD102-defined omental macrophages, and found that the CD102^+^ fraction of omental macrophages was enriched for TIM4^+^ cells (Figure 1e, left), and comprised the majority of TIM4^+^ omental macrophages (Figure 1e, right). Notably, P1 peritoneal and omental macrophages expressed equivalent levels of TIM4 (Figure 1d). Thus, omental CD102^+^ and CD102^−^ macrophages respectively largely encompass TIM4^+^ and TIM4^−^ populations described previously [15] and hence, we subdivide omental macrophages based on CD102 and F4/80 for the remainder of the study.

Finally, to ascertain if CD102-defined macrophage subsets inhabit discrete areas within the omentum, we used whole-mount immunofluorescence confocal microscopy. As the anti-CD102 and anti-F4/80 antibody clones suitable for immunofluorescence were both of rat origin, we stained for F4/80 alongside GATA6, which is uniformly expressed by CD102^+^ omental macrophages [6]. Abundant F4/80^+^ macrophages could be detected among omental adipocytes but these did not express GATA6 (Figure 1f). In contrast, GATA6^+^ F4/80^+^ macrophages were detected around the nuclei-dense omental FALC among numerous GATA6^+^ F4/80^−^ cells (Figure 1g, top, asterisk) that likely represent the surface mesothelium (Figure S1j). However, these macrophages were relatively rare and also accompanied by GATA6^–^F4/80^+^ cells (Figure 1g, top, hash symbol). Co-staining of GATA6 with TIM4 revealed a similar distribution of GATA6^+^TIM4^+^ and GATA6^−^TIM4^+^ cells around the FALC (Figure 1g, bottom, asterisks and hash symbol respectively).

Hence, the steady-state omentum is populated by several macrophage populations including FALC-associated cells that exhibit phenotypic similarity with resident peritoneal CD102^+^ macrophages.

### Origin-restricted migration of peritoneal macrophages to the omentum

**Figure 2:**
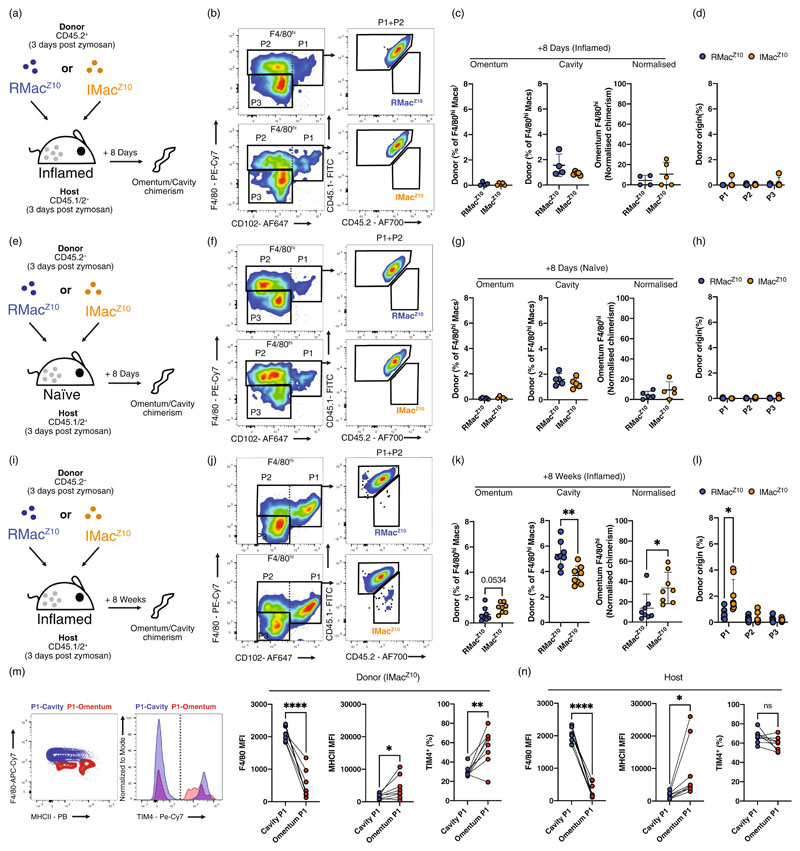
Inflammatory macrophages migrate to the omentum post resolution. (a) Experimental schematic. (b) Gating strategy to identify CD45.1^−^CD45.2^+^ donor macrophages within omental F4/80^hi^ (P1 and P2) macrophages 8 days post transfer into inflamed CD45.1^+^CD45.2^+^ recipient mice. (c) Proportion of F4/80^hi^ macrophages in the omentum, the cavity and the former normalized to the latter that are of donor origin 8 days (RMac^Z10^ *n* = 4, IMac^Z10^ *n* = 5) post transfer into inflamed recipients. (d) Proportion of omental P1, P2 or P3 cells that are of donor origin of samples shown in c. Statistical significance determined using multiple *t*-tests with Holm Sidak adjustment. (e) Experimental schematic. (f) Gating strategy to identify CD45.1^−^CD45.2^+^ donor macrophages within omental F4/80^hi^ (P1 and P2) macrophages 8 days post transfer into naïve CD45.1^+^CD45.2^+^ recipient mice. (g) Proportion of F4/80^hi^ macrophages in the omentum, the cavity and the former normalized to the latter that are of donor origin 8 days (*n* = 5/group) post transfer into naïve recipients. (h) Proportion of omental P1, P2 or P3 cells that are of donor origin of samples shown in g. Statistical significance determined using multiple t-tests with Holm Sidak adjustment. (i) Experimental schematic. (j) Gating strategy to identify CD45.1^−^CD45.2^+^ donor macrophages within omental F4/80^hi^ (P1 and P2) macrophages 8 weeks post transfer into inflamed CD45.1^+^CD45.2^+^ recipient mice. (k) Proportion of F4/80^hi^ macrophages in the omentum, the cavity and the former normalized to the latter that are of donor origin 8 weeks (*n* = 8/group) post transfer into inflamed recipients. (l) Proportion of omental P1, P2 or P3 cells that are of donor origin of samples shown in k. Statistical significance determined using multiple *t*-tests with Holm Sidak adjustment. (m) Representative expression and quantification of F4/80, MHCII and TIM4 on donor peritoneal and omental P1 cells (*n* = 8). Statistical significance was determined using paired student’s *t* test. (n) Quantification of F4/80, MHCII and TIM4 on host peritoneal and omental P1 cells (*n* = 8). Data presented was pooled from at least two independent experiments and is presented as mean ± standard deviation with symbols representing individual animals. Reanalysis of published data [19] is presented for peritoneal cells. *p* values are reported as *p < 0.05, **p < 0.01, ****p < 0.0001

To determine if migration occurs between the cavity and omentum and whether this is influenced by macrophage origin, we first assessed the period following resolution of mild peritoneal inflammation when abundant inflammation-elicited monocyte-derived and resident macrophages are found [19]. For this, we examined omenta from the same adoptive cell transfer experiments that we previously used to track the fate of these macrophage populations in the peritoneal cavity [19]. These experiments employed IP injection of low-dose zymosan A (10 μg/mouse), a well-established model of transient sterile neutrophilic peritoneal inflammation that largely resolves within 3 days [19,28]. To definitively distinguish recruited inflammatory macrophages from established resident cells, we used a combination of F4/80 expression and prior labelling of established resident macrophages by injection of the particulate dye PKH26-PCL 24 h before initiation of inflammation [19]. This dye is phagocytosed and retained by resident macrophages and effectively cleared from the cavity by the point of zymosan injection [18,19]. At day 3 post-inflammation, established resident macrophages were subsequently identified as F4/80^hi^ PKH26-PCL^+^ (termed RMac^Z10^) while recruited monocyte-derived inflammatory macrophages were F4/80^intermediate^ PKH26-PCL^−^ (IMac^Z10^; Figure S2a) [17,19]. For each population, 1 x 10^5^ cells were purified from CD45.2^+^ donor mice and transferred into the cavity of zymosan-treated equivalently-inflamed CD45.1/2^+^ recipient mice. Eight days later peritoneal cells were isolated and the omenta enzymatically digested and assessed for donor cell content by flow-cytometry (Figure 2a). Surprisingly, we found no quantifiable contribution of donor RMac^Z10^ or IMac^Z10^ to the omental F4/80^hi^ macrophage compartment at this stage (Figure 2b,c) even when these were subdivided into CD102^+^ (P1) and CD102^−^ (P2) populations (Figure 2b,d), nor were donor cells present within the omental CD11b^+^F4/80^–^ (P3; Figure 2b,d) or CD11b^–^ fractions (data not shown). In contrast, reanalysis of our published data for peritoneal lavage cells revealed that donor RMac^Z10^ and IMac^Z10^ [10] readily contributed to F4/80^hi^ macrophages in the cavity and accounted for between 1%–2% of these cells (Figure 2c), equating to the survival of approximately 40% of the transferred cells [19].

We postulated that the failure to detect migration could be a consequence of physiological alterations to the omentum arising from inflammation. Hence, we transferred donor RMac^Z10^ and IMac^Z10^ into naïve recipient mice (Figure 2e). Even in this setting, we did not detect significant contribution of donor cells to any omental myeloid compartment (Figure 2f–h), despite their persistence within the cavity F4/80^hi^ compartment (Figure 2g) [19]. To determine if migration might occur over a longer time-frame, we subsequently assessed frequencies of donor cells 8 weeks following transfer into equivalently-inflamed recipient mice (Figure 2i). In addition, to increase our ability to detect donor cells we injected 2 × 10^5^ cells of each population. However, we still could not confidently detect donor RMac^Z10^ within the omentum (Figure 2j–l). In contrast, a convincing population of omental IMac^Z10^-derived cells was detectable at this 8-week timepoint (Figure 2j–l) despite these cells persisting somewhat more poorly in the cavity than RMac^Z10^ (Figure 2k) in this set of experiments. Indeed, assessment of the frequency of donor IMac^Z10^ in omentum as a proportion of those in the cavity suggested that IMac^Z10^ had a greater propensity to migrate than RMac^Z10^ and that around 40% of omental macrophages were likely to have arisen from cavity-derived inflammatory macrophages over this period (Figure 2k). Notably, donor IMac^Z10^ were almost exclusively found within the omental CD102^+^ P1 subset of macrophages (Figure 2l; Figure S2b), of which close to 60% were seemingly of inflammatory peritoneal macrophage origin (Figure S2c). Donor IMac^Z10^ within the omental P1 compartment also expressed less F4/80 and more MHCII than their CD102^+^ peritoneal counterparts (Figure 2m) and similar to recipient cells in these sites (Figure 2n), suggesting that cavity-derived IMac^Z10^ adopted an omental phenotype upon migration. This was further emphasized by the greater proportion of donor IMac^Z10^ that expressed TIM4 in the omentum than cavity (Figure 2m), where they attained levels that were similar to host omental P1 macrophages (Figure 2m,n). Finally, adoptive transfer of 2 × 10^5^ naïve resident macrophages (RMac) into naïve recipient animals revealed that, similar to RMac^Z10^ in inflamed recipients, little migration of donor cells occurred over 8 weeks under non-inflamed conditions, although those few cells we did detect appeared to be within P1 (Figure S2d).

Taken together, these data suggest that established resident peritoneal macrophages are largely non-migratory, whereas monocyte-derived inflammatory macrophages migrate to the omentum, adopt an omental CD102^+^ P1-like phenotype, and comprise a large proportion of this population in the months following inflammation.

### Temporal depletion of omental macrophages allows migration of resident peritoneal cells

Next, we explored whether migration of established resident peritoneal macrophages is normally prevented by the presence of endogenous omental resident cells. Intraperitoneal administration of clodronate liposomes results in depletion of omental [29] and peritoneal macrophages [19,29,30]. We found injection of a comparatively low dose of clodronate liposomes (0.0625 mg) into the cavity completely depleted F4/80^hi^ peritoneal macrophages with no overt signs of peritoneal neutrophilia (Figure S2e–h) making the cavity amenable to adoptive transfer studies [19]. Even at this low dose, hepatic Kupffer cells were largely lost by 24 h and were reconstituted by TIM4^−^ monocyte-derived Kupffer cells [31] (Figure S2i). Similarly, 15 days after clodronate liposome injection the proportion of omental P1 and P2 macrophages that expressed TIM4, a proposed marker of embryonic omental macrophages [15], had decreased (Figure S2j), indicating some depletion. Hence, we postulated that this regimen would create availability within the omental ‘niche’ and allow for migration of peritoneal cells, should such migratory route exist.

**Figure 3:**
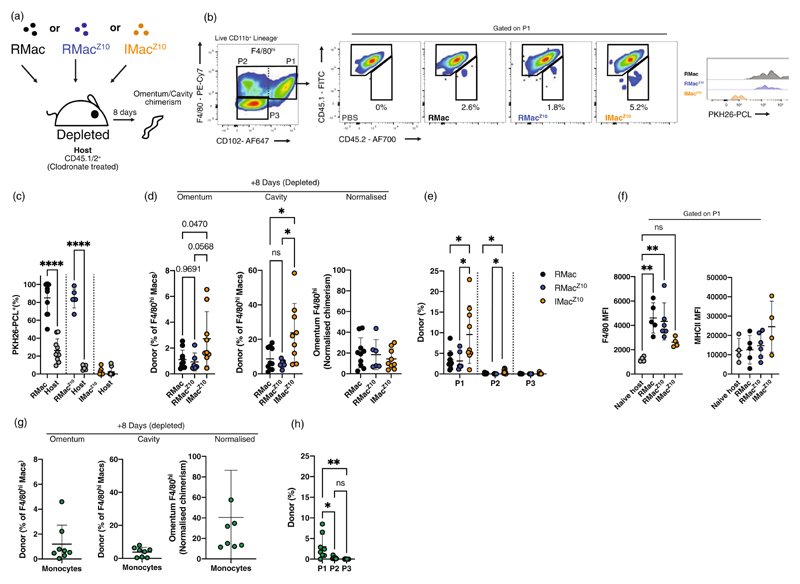
Peritoneal cavity macrophages migrate to macrophage-deplete omentum. (a) Experimental schematic. (b) Gating strategy to identify CD45.1^−^CD45.2^+^ donor macrophages within omental F4/80^hi^ P1 macrophages and representative histogram of PKH26-PCL labelling on donor macrophages, 8 days following transfer into clodronate-depleted recipient mice. (c) Proportion of P1 donor RMac (black; n = 10), RMac^Z10^ (blue; *n* = 6) or IMac^Z10^ (orange; *n* = 11) or host cells that are PKH26-PCL^+^. Statistical significance determined using oneway ANOVA with Sidak multiple comparisons adjustment. (d) Proportion of F4/80^hi^ macrophages in the omentum, the cavity and the former normalized to the latter that are of donor origin 8 weeks post transfer into clodronate-depleted recipients. (RMac, black, *n* = 10; RMac^Z10^, blue, *n* = 6; IMac^Z10^, orange, *n* = 11). Statistical significance determined using one-way ANOVA with Tukey’s multiple comparisons test. (e) Proportion of omental P1, P2 or P3 cells that are of donor origin of samples shown in d. Statistical significance determined using one-way ANOVA with Sidak multiple comparisons adjustment. (f) Mean fluorescence intensity for F4/80 and MHCII on P1 omental donor cells (RMac, black, *n* = 10; RMac^Z10^, blue, *n* = 6; IMac^Z10^, orange, *n* = 11) compared to that of naïve host omentum P1 (*n* = 3). Statistical significance determined using one-way ANOVA with Tukey’s multiple comparisons test. (g) Proportion of F4/80^hi^ macrophages in the omentum, the cavity and the former normalized to the latter are of donor origin 8 days following transfer of Ly6C^hi^ monocytes (*n* = 8). (h) Proportion of omental P1, P2 or P3 cells that are of donor origin of samples shown in g. Statistical significance determined using one-way ANOVA with Tukey’s multiple comparisons test. Data presented was pooled from at least two independent experiments and is presented as mean ± standard deviation with symbols representing individual animals. Reanalysis of published data [19] is presented for peritoneal cells in a-f. *p* values are reported as *p < 0.05, **p < 0.01, ****p < 0.0001

Eight days following transfer of RMac, RMac^Z10^ or IMac^Z10^ into macrophage-depleted mice (Figure 3a), an appreciable population of CD45.2^+^ donor cells could be detected within the omental F4/80^hi^ macrophage compartment (Figure 3b,c). Consistent with labelling at the time of transfer (Figure S2a) [19], RMac and RMac^Z10^ in the omentum were almost exclusively PKH26-PCL^+^ whereas both host macrophages and donor IMac^Z10^ were PKH26-PCL^−^ (Figure 3b,c), confirming the identity of these cells. Although donor IMac^Z10^ appeared to migrate to the omentum somewhat better (Figure 3d, lefthand graph), they also more readily expanded in the peritoneal cavity under these conditions (Figure 3d, middle graph) [19] and normalization of donor frequency in the omentum to that in the cavity suggested all three donor populations exhibited equivalent migratory capacity (Figure 3d, righthand graph). Irrespective of the population transferred, donor cells were almost exclusively found within the P1 CD102^+^ omental macrophage compartment, although IMac^Z10^ seemingly also contributed marginally to the P2 CD102^−^ population (Figure 3e). Notably, unlike IMac^Z10^-derived cells, omental RMac and RMac^Z10^ exhibited higher expression of F4/80 than omental P1 macrophages from nondepleted mice, reminiscent of their peritoneal phenotype. In contrast, all donor populations exhibited high levels of MHCII expression characteristic of omental P1 macrophages (Figure 3f), unlike in the cavity where RMac and RMac^Z10^ remain largely MHCII^−^ [19]. The same pattern in of results was apparent at week 8 post-transfer except that omental RMac appeared to down-regulate F4/80 expression by this time (Figure S3a–d). However, caution should be taken with interpretation of these data as very few donor RMac and RMac^Z10^ could be detected at this time point and PKH26-PCL dye-label was no longer present in these cells in the omentum or the more numerous populations in cavity and could not be used to confirm donor identity (data not shown). Hence, in the absence of endogenous omental macrophages, established resident peritoneal macrophages migrate to the omentum and adopt features associated with omental P1 macrophages but, at least transiently, these cells also retain characteristics of cavity cells that delineate them from steady-state omental macrophages.

### Divergent repopulation of peritoneal and omental niches by resident and inflammatory macrophages

The near-complete lack of contribution of any donor population to omental P2 cells is intriguing. As the P2 phenotype is unique to the omentum (Figure 1a), it is possible that RMac or IMac^Z10^ lack the plasticity required to contribute. Given monocytes retain greater plasticity than differentiated macrophages [32], we purified the abundant Ly6C^hi^ monocytes found within the cavity at 4 h post-zymosan administration (Figure S3e) [19] and transferred these into clodronate-depleted recipients. Transferred monocytes contributed to the F4/80^hi^ macrophage compartments in both the cavity and omentum (Figure 3g) with similar efficacy to resident macrophages in our earlier experiments (Figure 3d). Monocyte-derived cells were also almost wholly restricted to the P1 omental macrophage compartment (Figure 3h). The failure of transferred peritoneal cells, irrespective of origin, to contribute to omental P2 macrophages in this system suggests a non-peritoneal origin or inefficient/transient depletion of this population using clodronate liposomes rather than a limitation in plasticity of peritoneal macrophages.

To confirm whether peritoneal macrophages can repopulate the omental F4/80^+^CD102^−^ (P2) niche, and examine in more depth the functional outcome of the difference in repopulation capacity of the resident and recruited populations [19] (Figure 3d), we looked for a system in which competition for the cavity and omental niches by endogenous cells was comprehensively lacking. Hence, we examined a recently-developed mouse line that lacks the fms-intronic regulatory element (FIRE) super enhancer of the *Csf1r* gene. These *Csf1r*^ΔFIRE/ΔFIRE^ mice lack Colony Stimulating Factor 1 Receptor (CSF1R) expression on numerous monocyte/macrophage populations and are consequently devoid of resident F4/80^hi^ peritoneal macrophages, although they retain normal frequencies of peritoneal F4/80^lo^ myeloid cells [20]. Analysis of *Csf1r*^ΔFIRE/ΔFIRE^ mice confirmed an almost complete lack of cavity CD11b^+^ myeloid cells that resulted from the absence of F4/80^hi^ macrophages (Figure S4a–c) without concurrent increase in F4/80^lo^ myeloid cells or neutrophils and only marginal increase in Ly6C^+^ monocytes (Figure S4c). Furthermore, omenta from *Csf1r*^ΔFIRE/ΔFIRE^ mice contained approximately a third of the normal complement of CD11b^+^ cells (Figure S4d,e) arising from an almost complete absence of CD102^+^ and CD102^−^F4/80^hi^ macrophages (Figure S4d,e) whereas the complement of F4/80^lo^ cells, including Ly6C^+^ monocytes, and neutrophils was unaffected (Figure S4e). Hence, *Csf1r*^ΔFIRE/ΔFIRE^ mice are devoid of both cavity and omental F4/80^hi^ macrophages without overt compensatory increase in monocytes, F4/80^lo/−^ myeloid cells, or neutrophils, providing an ideal system to test the repopulation capacity of peritoneal cells in both tissue sites.

**Figure 4:**
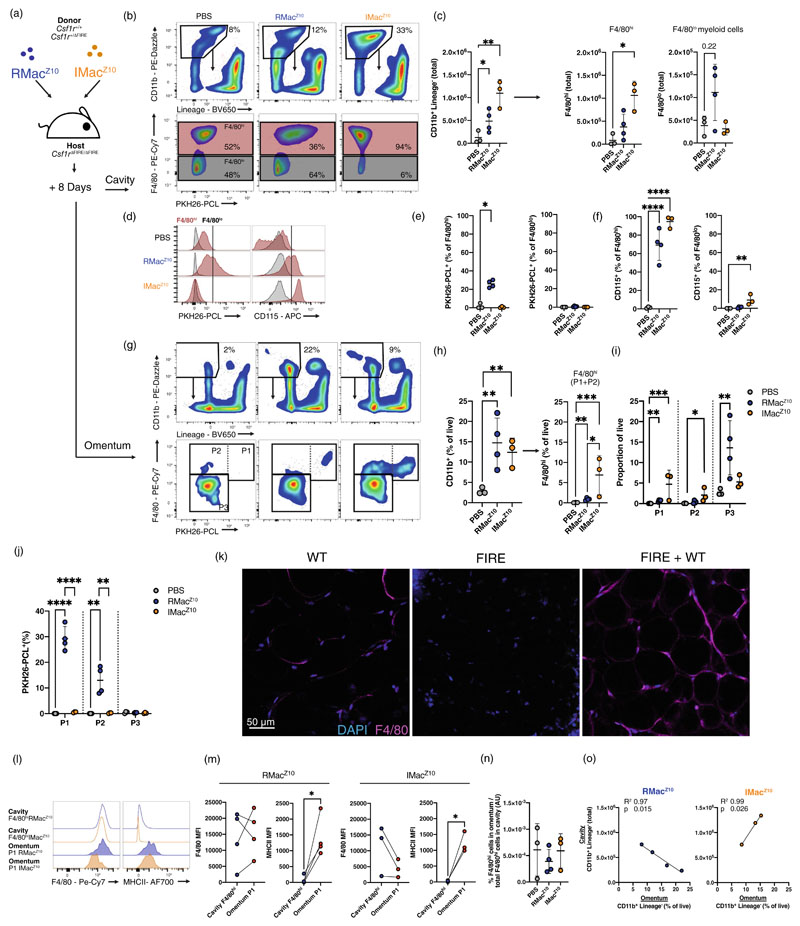
Resident and inflammatory macrophages differ in capacity to repopulate peritoneal and omental niches. (a) Experimental schematic. (b) Gating strategy to identify F4/80^hi^ and F4/80^lo^ macrophages in the peritoneal cavity following transfer of RMac^Z10^ or IMac^Z10^ from *Csf1r*^+/+^*/Csf1r*^+^^/ΔFIRE^ mice or PBS into *Csf1r*^ΔFIRE/ΔFIRE^ recipient mice. (c) Total CD11b^+^Lineage^−^ myeloid cells and F4/80^hi^ and F4/80^lo^ macrophages in the cavity following transfer of PBS (*n* = 3), RMac^Z10^ (*n* = 4)or IMac^Z10^ (*n* = 3). Statistical significance determined using one-way ANOVA with Tukey’s multiple comparisons test. (d) Representative histogram of PKH26-PCL and CD115 expression of cavity F4/80^hi^ and F4/80^lo^ macrophages in the peritoneal cavity. (e) Proportion of cavity F4/80^hi^ and F4/80^lo^ macrophages that are PKH26-PCL^+^ 8 days post transfer of indicated populations. (f) Proportion of cavity F4/80^hi^ and F4/80^lo^ macrophages that are CD115^+^ 8 days post transfer of indicated populations. Statistical significance determined using one-way ANOVA with Tukey’s multiple comparisons test. (g) Gating strategy to identify omental F4/80^hi^ omental macrophages and subdivide into CD102^+^ (P1) and CD102^−^ (P2) subsets. (h) Proportion of live omental cells that are CD11b^+^Lineage^−^ myeloid cells and F4/80^hi^ and macrophages following transfer of PBS (n = 3), RMac^Z10^ (*n* = 4) or IMac^Z10^ (*n* = 3) Statistical significance determined using one-way ANOVA with Sidak multiple comparisons adjustment. (i) Proportion of live omental cells that are P1, P2 or P3 cells in samples shown in h. Statistical significance determined using one-way ANOVA with Sidak multiple comparisons adjustment. (j) Proportion of omental cells that are PKH26-PCL^+^ following transfer of indicated populations. Statistical significance determined using one-way ANOVA with Sidak multiple comparisons adjustment. (k) Representative confocal imaging of *Csf1r*^ΔFIRE/ΔFIRE^ omental adipose tissue 8 days post transfer of the complete *Csf1r*^+/+^ peritoneal exudate cell compartment at day 3 post zymosan. (l) Representative F4/80 and MHCII intensity on indicated populations. (m) Mean fluorescence intensity for F4/80 and MHCII on cavity F4/80^hi^ and P1 omental macrophages following transfer of indicated populations. Statistical significance determined using paired student’s *t* test. (n) Normalized engraftment of indicated populations in arbitrary units. Normalize engraftment was calculated as F4/80^hi^ omental macrophages (% of live)/F4/80^hi^ cavity macrophages (total) × 100. (o) Correlation between total CD11b^+^Lineage^−^ cells in the peritoneal cavity (y-axis) and the proportion of live cells that are CD11b^+^Lineage^−^ in the omentum (*x*-axis) following transfer of RMac^Z10^ or RMac^Z10^. Correlation was assessed using a simple linear regression. Flow-cytometric data is presented as mean ± standard deviation with symbols representing individual animals (PBS, *n* = 3; RMac^Z10^, *n* = 4; IMac^Z10^, *n* = 3) and was pooled from four independent experiments. *p* values are reported as *p < 0.05, **p < 0.01, ***p < 0.001, ****p < 0.0001

Similar to our earlier experiments, we FACS-purified 1 × 10^5^ RMac^Z10^ or IMac^Z10^ but sourced from *Csf1r*^+/-^^ΔFIRE^ or *Csf1r*^+/+^ littermates 3 days after zymosan injection (Figure S4f) and transferred these into *Csf1r*^ΔFIRE/ΔFIRE^ recipients (Figure 4a). Zymosan injection induced a similar response in both *Csf1r*^+/ΔFIRE^ and *Csf1r*^+/+^ littermates (Figure S4f), and hence, to source sufficient cells for transfer we used donor cells from *Csf1r*^+/ΔFIRE^ and *Csf1r*^+/+^ littermates or a mix of both. By day 8 post-transfer, a greater number of CD11b^+^ myeloid cells were found in the cavity of recipient *Csf1r*^ΔFIRE/ΔFIRE^ mice than in those given vehicle control, irrespective of the population transferred (Figure 4b,c). For RMac^Z10^, this represented a 4-fold increase over the number transferred. However, consistent with the degree of engraftment we reported in clodronate-depleted mice [19], IMac^Z10^ seemingly expanded more efficiently (Figure 4c), increasing more than 10-fold and generating similar numbers to those normally found in *Csf1r*^+/+^ animals (Figure 4c vs. Figure S4c). Critically, transfer of IMac^Z10^ led to an exclusive increase in F4/80^hi^ resident peritoneal macrophages (Figure 4c) whereas the increase in CD11b^+^ cells following transfer of RMac^Z10^ seemingly comprised both F4/80^hi^ and CD11b^+^F4/80^lo^ myeloid compartments (Figure 4c). Correlation of the number of F4/80^hi^ and F4/80^lo^ myeloid cells post-transfer indicated that incomplete reconstitution of the cavity F4/80^hi^ population was associated with increased numbers of F4/80^lo^ macrophages (Figure S4g), and demonstrated that the only animal to receive RMac^Z10^ that did not exhibit high numbers of F4/80^lo^ cells had uniquely high levels of F4/80^hi^ cells for this group. Using expression of CD115 (CSF1R), which is absent on *Csf1r*^ΔFIRE/ΔFIRE^ recipient cells [20] (Figure 4d), and PKH26-PCL dye labelling, which is restricted to transferred RMac^Z10^ (Figure S4f), we confirmed that the expanded F4/80^hi^ macrophage compartment in recipient mice largely comprised donor CD115^+^PKH26-PCL^+^ RMac^Z10^ or CD115^+^IMac^Z10^ cells (Figure 4d-f). In contrast, CD115 expression indicated that only transferred CD115^+^IMac^Z10^ contributed directly to the CD11b^+^F4/80^lo^ myeloid compartment and only to a very limited degree whereas CD115^+^PKH26-PCL^+^RMac^Z10^ did not (Figure 4f). Hence, the CD11b^+^F4/80^lo^ cells detected following transfer of RMac^Z10^ are of recipient origin and consistent with this, a large proportion were found to express the monocyte marker Ly6C (Figure S4h). Notably, transfer of RMac^Z10^ or IMac^Z10^ expanded the peritoneal eosinophil compartment beyond that of vehicle treated controls (Figure S4i), mirroring the role of peritoneal macrophages during helminth infection [33,34]. Hence, RMac^Z10^ and IMac^Z10^ have similar capacity to regulate peritoneal eosinophils but differ in ability to rapidly replenish the peritoneal macrophage compartment.

Next, analysis of the omenta revealed that transfer of either RMac^Z10^ or IMac^Z10^ led to the overall expansion of the omental CD11b^+^Lineage^−^ compartment (Figure 4g, h). However, following transfer of RMac^Z10^, this increase was overwhelmingly due to expansion of omental CD11b^+^CD102^−^F4/80^lo^ myeloid cells (Figure 4i) that appeared largely of recipient origin as few were positive for PKH26-PCL (Figure 4j) and most expressed Ly6C (Figure S4h). In contrast, the expansion of CD11b^+-^ Lineage^−^ cells following transfer of IMac^Z10^ was almost exclusively due to an increase in F4/80^hi^ omental macrophages (Figure 4h) through modest expansion of both CD102^+^ P1 and CD102^−^ P2 populations (Figure 4i). Confocal microscopy confirmed that transfer of peritoneal cells isolated at day 3 post zymosan injection led to efficient repopulation of omental adipose macrophages (Figure 4k) Notably, as we had observed in macrophage-depleted mice at this timepoint, the few omental P1 cells derived from RMac^Z10^ adopted the omental MHCII^hi^ phenotype but retained similar expression of F4/80 to their peritoneal counterparts (Figure 4l,m), whereas there was a trend for omental P1 cells derived from IMac^Z10^ to adopt intermediate expression of F4/80 as well as higher expression of MHCII characteristic of omental P1 macrophages. Again, normalizing the frequency of P1 and P2 F4/80^hi^ macrophages in the omentum with their frequency in the cavity indicated that the increased contribution of IMac^Z10^ to omental macrophages correlated with their abundance the cavity (Figure 4n). Likewise, following transfer of IMac^Z10^ the number of CD11b^+^ cells in the cavity directly correlated with the proportion in the omentum (Figure 4o). These data suggest that enhanced migration of IMac^Z10^ may, in part, result from their greater expansion in the cavity. Conversely, in mice given RMac^Z10^, the number of CD11b^+^ cavity myeloid cells negatively correlated with those in the omentum (Figure 4o) suggesting the inability of RMac^Z10^ to expand drives infiltration of recipient omental P3 myeloid cells. Importantly, despite utilizing distinct mechanisms to repopulate available niche space, transfer of either RMac^Z10^ or IMac^Z10^ appeared to reconstitute the omental CD11b^+^ Lineage^−^ niche of *Csf1r*^ΔFIRE/ΔFIRE^ to levels normally found in *Csf1r*^+/+^ littermates (Compare Figure 4h to Figure S4e). Hence, these data highlight further functional differences between RMac^Z10^ and IMac^Z10^, namely a propensity of inflammatory macrophages to more rapidly repopulate tissues and acquire tissue-specific phenotype, including omental P1 and P2 macrophages, while resident macrophages have more limited repopulation capacity and instead drive recruitment of monocyte-derived cells.

## Discussion

Phagocyte migration between the peritoneal cavity and omentum has long been hypothesized but has only been shown decisively to occur during inflammation, whereupon resident peritoneal macrophages rapidly migrate en masse to omental FALCs to promote FALC-development [14,17,35]. Here, we describe a second pathway of peritoneal to omental migration that is selective for monocyte-derived resident cells and occurs gradually over the months that follow peritoneal inflammation.

In line with recent publications [36,37] we demonstrate that the murine omentum contains a population of CD102^+^ macrophages that, like those in the cavity [4,21], are CSF1R^+^ and rely on CSF1R signalling for survival. By virtue of their expression of peritoneal macrophage-associated transcription factor GATA6 [6], we demonstrate CD102^+^ omental macrophages to be associated with omental FALCS. These cells represented the majority of omental TIM4^+^ macrophages and hence, appear to align with the long-lived FALC-associated CSF1-dependent TIM4^+^ omental macrophages described recently by Etzerodt and colleagues [15].

Despite phenotypic similarities between cavity and FALC-associated CD102^+^ macrophages, we found little evidence of routine migration of established resident CD102^+^ peritoneal macrophages to the omentum under non-inflammatory conditions, pre- or post-inflammation. These data seemingly fit the long-standing view that peritoneal macrophages arise from omental cells rather than vice-versa [6]. However, the efficient contribution of inflammatory peritoneal macrophages to CD102^+^ omental macrophages following resolution of inflammation suggests inflammation fundamentally alters this relationship or monocyte-derived resident peritoneal macrophages have a unique migratory function. Either way, these data reveal previously unappreciated functional heterogeneity within the post-resolution peritoneal cavity macrophage compartment governed by cell origin. Notably, monocyte-derived resident peritoneal macrophages recruited under steady-state conditions exhibit striking phenotypic, functional and transcriptional similarities to those that persist following mild inflammation [19] yet comprise only 10%–15% of the resident F4/80^hi^ macrophages in young adult mice [5]. Hence, it remains possible that at least part of the CD102^+^ population in healthy omental tissue arise from migration of these pre-established monocyte-derived resident peritoneal macrophages but that transfer of resident peritoneal macrophages en bloc was not sensitive enough to detect this.

Relatively little migration of inflammatory macro-phages occurred within the first week post-transfer into resolution phase or naïve mice compared with the levels detectable at 8 weeks post-inflammation. These kinetics suggest migration through the omentum is not requisite for the differentiation of inflammatory macrophages into long-lived F4/80^hi^ CD102^+^ resident peritoneal macrophages that occurs within this initial period [19] and that the bulk of migration occurs thereafter. Whether inflammatory macrophages acquire a migrative capacity after further differentiating in the cavity, or if migration occurs gradually and cumulatively resulting in greater detection at week 8 remains unclear, but inflammatory macrophage-derived resident cells continue to express higher levels of chemokine receptors for many months after inflammation [19], including CCR5 and CCR2 for which the omentum is a source of ligands [26,38]. Greater emigration of inflammatory macrophages could explain why these cells, despite exhibiting higher rates of proliferation, do not outcompete established resident macrophages in the months following inflammation [19].

The migration of resident peritoneal macrophages in macrophage-depleted mice suggests incumbent omental macrophages may normally prevent this. Although transfer into a macrophage-deficient environment could result in activation-induced migration of resident peritoneal macrophages, we optimized our liposome-depletion system to limit inflammation, as evidenced by the lack of neutrophilia. Similarly, naïve *Csf1r*^ΔFIRE/ΔFIRE^ mice exhibited normal numbers of cavity and omental neutrophils suggesting the absence of inflammatory stimuli. Hence, we propose the peritoneal cavity and omental FALC comprise a partially overlapping macrophage niche, access to which appears dictated by origin, inflammatory status, and competition from established omental cells.

We also found abundant macrophages within omental adipose tissue that were GATA6^−^ and likely represent the bulk of the CD102^−^F4/80^+^ cells identified by flow cytometry. While not normally replenished from the cavity pre or post inflammation, these cells share access to peritoneal fluid as evidenced by uptake of fluorescent molecules injected intraperitoneally, and can be repopulated by cavity monocyte-derived macrophages when depleted. Hence, a partial overlap in niche also appears to exist between omental adipose macrophages and peritoneal cells. The relevance of these findings is expounded by the development of approaches to deplete omental macrophages during peritoneal disease while leaving peritoneal macrophages intact [15].

Whether migration occurs from omentum to cavity and whether cells that migrate to the omentum subsequently remain long-term or are continually replaced from the cavity remains unclear. Indeed, the elevated migration of inflammatory macrophages in macrophage-depleted mice could underlie their more rapid transition to a GATA6^hi^ resident peritoneal phenotype in the cavity of these mice [19]. However, a main tenet supporting the hypothesis that peritoneal macrophages arise in the omentum comes from the accumulation of GATA6-deficient monocyte-derived macrophages in omenta of macrophage-specific GATA6 knock-out mice, as this suggests these cells require GATA6 to emigrate from omentum to cavity [6]. Given inflammatory peritoneal macrophages express less GATA6 than established resident cells for months post-inflammation and a transcriptional identity akin to GATA6-deficient resident peritoneal macrophages [19], our observations suggest GATA6-deficient macrophages may actually accumulate in the omentum due to an increased propensity to migrate from the cavity. Hence, reassessment of the role of omental macrophages in generation of peritoneal macrophages is warranted.

The phenotypic differences between omental and cavity CD102^+^ macrophages imply discrete programming signals within these sites. For example, omental FALC seemingly programme for heightened MHCII expression irrespective of macrophage origin, aligning with a primary role of FALC in lymphocyte regulation [2]. Notably, supernatant from cultured omentum downregulates MHCII expression by inflammatory macrophages *in vitro* [19], but given our current findings it seems likely that omental factors extrinsic to FALC may be responsible for this. Since performing most of our study, CD163 was found to be expressed by omental FALC-associated macrophages but largely absent on cavity macrophages [15] yet, similar to MHCII, expression appears unaffected by soluble omentum factors [8] suggesting FALC-specific regulation. Interestingly, although TIM4 has been proposed as marker of embryonic macrophages across tissues [39], including the omentum [15], the omental niche appeared competent at driving expression of this molecule on monocyte-derived macrophages following inflammation. Although our understanding of the transcriptional similarity of peritoneal and omental macrophages and consequently the degree to which peritoneal macrophages can acquire the identity of resident omental cells is incomplete, established resident peritoneal macrophages were less able than inflammatory cells to acquire the F4/80^intermediate^ phenotype of omental CD102^+^ cells, at least in the short-term. These data support the hypothesis that macrophages progressively lose the ability to acquire new identity as they differentiate [32,40,41]. Unfortunately, the poor detection of RMac at 8 weeks in the omenta of macrophage-depleted mice means we cannot be certain whether these cells persist long-term and eventually adopt the omental F4/80^intermediate^ phenotype. However, the presence of monocyte-derived cells within established CD102^+^ resident peritoneal macrophages [4,5] would nevertheless confound interpretation of these data and future examination of the plasticity of resident peritoneal macrophages will rely on our ability to separately fate-map those of recent monocyte origin from the embryonic/established population.

Just as inflammatory macrophages are better able than resident cells to repopulate a transiently-depleted niche [19], we now show that even in the complete absence of endogenous macrophages, established resident cells lack the rapid re-population capacity of recent recruits. Further investigation into the dynamics of proliferation, death and survival is needed to understand these differences but, elevated proliferative activity appears a common feature of recruited macrophages across tissues [42,43] including under non-inflamed conditions [4,5,19]. Notably, our findings that transfer of resident macrophages into *Csf1r*^ΔFIRE/ΔFIRE^ mice led to infiltration of monocytes into the omentum and probably the cavity also, and that this may be related to the inability of resident cells to rapidly repopulate the cavity autonomously mirror the proliferation of resident macrophages and concurrent recruitment of monocytes that occurs upon administration of excess CSF1 [44]. These data are consistent with a model whereby at suboptimal population density, established resident peritoneal macrophages both proliferate and drive recruitment of monocytes [45]. Given that transferred resident macrophages were the only cavity cells in recipient *Csf1r*^ΔFIRE/ΔFIRE^ mice to express appreciable surface CSF1R, our data suggest resident macrophages are indispensable to sense niche fullness likely through detection of cavity CSF1. Furthermore, our data predict that repopulation of a depleted niche by recruited macrophages results in more rapid restoration of homeostasis, with implications for targeting of monocytes during inflammatory diseases.

Taken together, these data highlight important functional differences in macrophage populations in the peritoneal cavity, and support a model of an overlapping cavity-omentum niche for migratory monocyte-derived macrophages. These findings are relevant to understanding the impact of inflammation in the peritoneal cavity and have importance for diseases in which omental macrophages are critical such as metastatic spread of abdominal cancer [15,36,46,47].

## Supplementary Material

Supplementary Information

## Data Availability

Data will be made available from the corresponding author upon reasonable request.

## Abbreviations

BM: bone marrow
CSF1: Colony Stimulating Factor 1
CSF1^AF647^: porcine CSF1 conjugated to Alexafluor 647
CSF1R: Colony Stimulating Factor 1 Receptor
FALC: fat-associated lymphoid cluster
IMac^Z10^: inflammatory macrophages 3 days post-inflammation
RMac: resident macrophages from naïve mice
RMac^Z10^: resident macrophages 3 days post-inflammation
