# Supplementary material for: Cell origin and niche availability dictate the capacity of peritoneal macrophages to colonize the cavity and omentum
Source: Immunology. Author manuscript; Available in PMC 2022 Sep 7. (PMC7613338; doi:10.1111/imm.13483)
Supplement: Supplementary Information [file EMS152654-supplement-Supplementary_Information.pdf]

SUPPORTING INFORMATION

Cell origin and niche availability dictate the capacity of peritoneal macrophages to colonize the cavity and omentum.

Pieter A Louwe, Stuart J Forbes, Cecile Benezech, Clare Pridans, Stephen J Jenkins

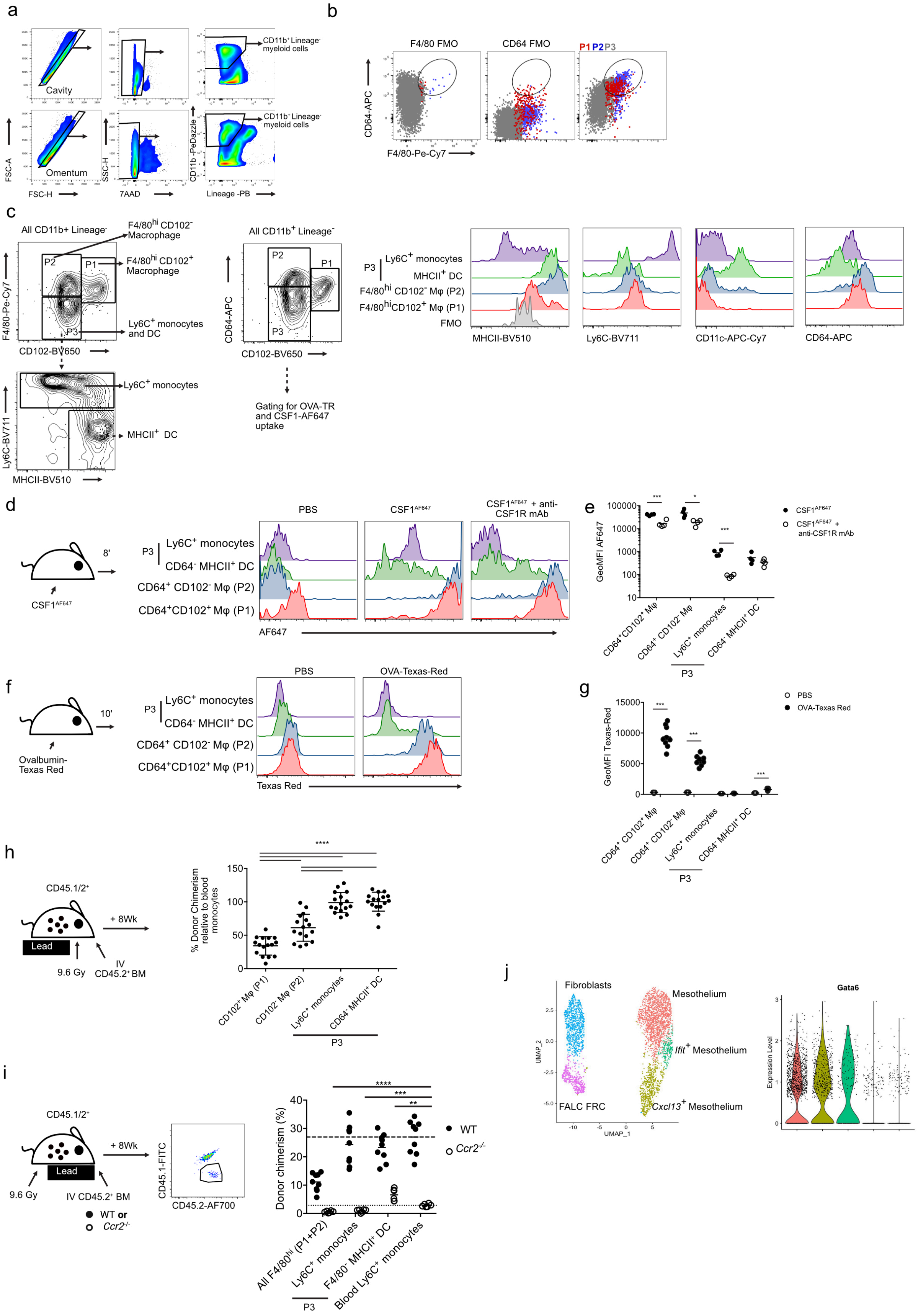

a
Cavity
Omentum
FSC-A
FSC-H
SSC-H
7AAD
CD11b -PeDazzle
Lineage -PB
CD11b+ Lineage- myeloid cells
b
F4/80 FMO
CD64 FMO
P1 P2 P3
CD64-APC
F4/80-Pe-Cy7
c
All CD11b+ Lineage-
F4/80hi CD102- Macrophage
F4/80hi CD102+ Macrophage
Ly6C+ monocytes and DC
P1
P2
P3
F4/80-Pe-Cy7
CD102-BV650
Ly6C+ monocytes
MHCII+ DC
Ly6C-BV711
MHCII-BV510
CD64-APC
Gating for OVA-TR and CSF1-AF647 uptake
F4/80hi CD102- Mφ (P2)
F4/80hiCD102+ Mφ (P1)
FMO
Ly6C-BV711
CD11c-APC-Cy7
d
8'
CSF1AF647
PBS
CSF1AF647 + anti-CSF1R mAb
Ly6C+ monocytes
CD64- MHCII+ DC
CD64+ CD102- Mφ (P2)
CD64+CD102+ Mφ (P1)
AF647
e
GeoMFI AF647
100000
10000
1000
100
10
CSF1AF647
CSF1AF647 + anti-CSF1R mAb
CD64+CD102+ Mφ
CD64+ CD102- Mφ
Ly6C+ monocytes
CD64- MHCII+ DC
f
10'
Ovalbumin-Texas Red
OVA-Texas-Red
Texas Red
g
GeoMFI Texas-Red
15000
10000
5000
0
OVA-Texas Red
h
CD45.1/2+
+ 8Wk
Lead
9.6 Gy
IV CD45.2+ BM
% Donor Chimerism relative to blood monocytes
150
50
CD102+ Mφ (P1)
CD102- Mφ (P2)
Ly6C+ monocytes
CD64- MHCII+ DC
i
CD45.1-FITC
CD45.2-AF700
WT or
Ccr2-/-
Donor chimerism (%)
40
30
20
WT
All F4/80hi (P1+P2)
F4/80- MHCII+ DC
Blood Ly6C+ monocytes
j
Fibroblasts
Mesothelium
Ifit+ Mesothelium
Cxcl13+ Mesothelium
FALC FRC
UMAP_1
UMAP_2
Gata6
Expression Level

Supplementary Figure 1. Comparative phenotype of steady-state peritoneal and omental macrophages.

(a) Representative gating strategy to identify myeloid cells in the peritoneal cavity (top) and the omentum (bottom).

(b) Representative F4/80 and CD64 expression of indicated omental myeloid cell subsets.

(c) Representative gating strategy to identify peritoneal macrophage subsets based on F4/80 (left) or CD64 (right) and CD102, Ly6C, and CD11c expression, and representative histograms for key markers on these cells.

(d) Experimental outline and representative fluorescence histogram for $CSF1^{AF647}$ treatment.

(e) Quantification of data shown in e (n=4/group).

(f) Experimental outline and representative fluorescence histogram for Ovalbumin Texas Red treatment.

(g) Quantification of data shown in f (n=9/group).

(h) Experimental outline for generation of tissue-protected bone marrow chimeras (n=16) and frequency of donor cells in omental myeloid subsets relative to that in Ly6C+ blood monocytes. This data has been re-analysed from previously published data from our laboratory. **(i)** Experimental outline for tissue-protected bone marrow chimeras reconstituted with WT (n=9) or $Ccr2^{-/-}$ BM (n=5) and frequency of donor cells in omental myeloid subsets.

(j) tSNE and *Gata6* mRNA expression in $CD45^{-}$ non-immune omental cells. Data derived from a previously published single-cell RNAseq dataset[23]. FRC, fibroblastic reticular cells.

Data presented was pooled from or is representative of at least two independent experiments and is presented as mean ± standard deviation with symbols representing individual animals. *p* values are reported as *p<0.05, **p<0.01, ***p<0.001, ****p<0.0001.

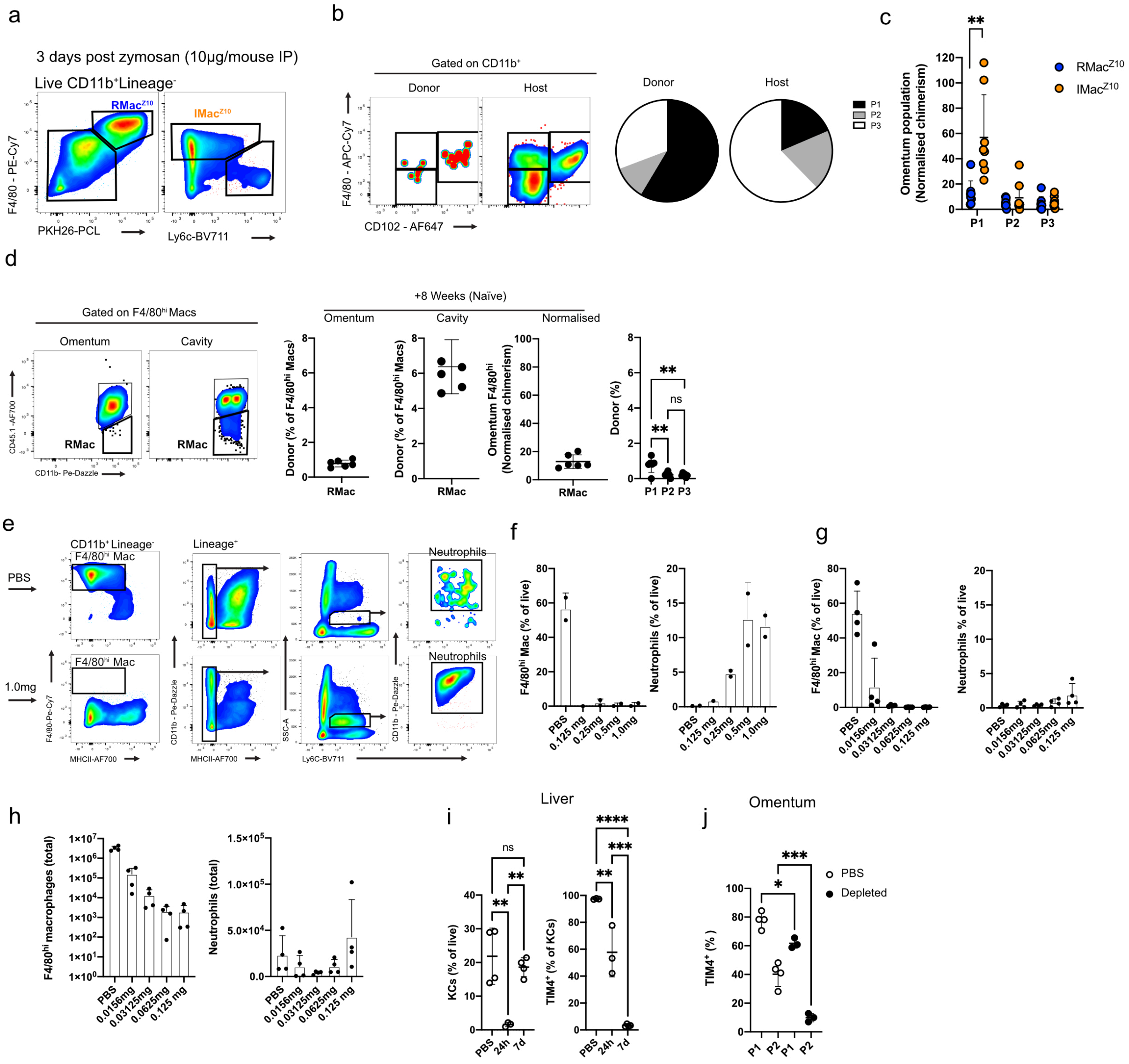

Supplementary Figure 2. Inflammatory macrophages migrate to the omentum post resolution.

(a) Representative gating strategy to identify PKH26-PCL^{+} RMac^{Z10} and PKH26-PCL^{-} IMac^{Z10} 3 days post zymosan. This plot was sourced from a previously published dataset[19].

(b) Donor (left) or host (right) omental P1, P2 and P3 myeloid subsets 8 weeks following transfer, and pie charts depicting the mean composition of donor cells.

(c) Proportion of P1, P2 and P3 cells in the omentum that are of donor origin normalised to their contribution to the F4/80^{hi} cavity compartment (n=8). Statistical significance determined using repeated t-test with Holm Sidak multiple comparisons adjustment.

(d) Proportion of F4/80^{hi} macrophages in the omentum and cavity, and the former normalised to the latter, that are of donor origin 8 weeks (n=8/group) post transfer of RMac into naive recipients (n=6). Statistical significance determined using one-way ANOVA with Tukey's multiple comparisons test.

(e) Representative gating to identify Lineage$^-$ peritoneal F4/80^{hi} macrophages (left) and Lineage$^+$ neutrophils (right) 7 days following ip injection of PBS or 1mg of clodronate liposomes.

(f) Proportion of live cells in the peritoneal cavity that are F4/80^{hi} macrophages or neutrophils 7 days following ip injection the indicated quantity of clodronate liposomes.

(g) Proportion of live cells in the peritoneal cavity that are F4/80^{hi}Macrophages or neutrophils 7 days following ip injection the indicated quantity of clodronate liposomes (n=4/group).

(h) Absolute number of F4/80^{hi} Macrophages or neutrophils of samples shown in **(g)**.

(i) Proportion of live liver cells that are CD11b^{lo} F4/80^{hi} Kupffer cells (KCs) and the proportion of KCs that express TIM4 following PBS (n=4) treatment or 24 hours (n=3) / 7 days (n=4) post treatment with 0.0625mg/mouse clodronate liposomes. Statistical significance determined using one-way ANOVA with Tukey's test for multiple comparisons.

(j) Proportion of each omental myeloid subset that expressed TIM4 15 days post treatment with PBS (n=4) or 0.0625mg clodronate liposomes (n=3). Statistical significance determined using one-way ANOVA with Tukey's test for multiple comparisons.

Data presented was pooled from at least two independent experiments, except d which was from a single experiment, and is presented as mean ± standard deviation with symbols representing individual animals. *p* values are reported as *p<0.05, **p<0.01, ***p<0.001, ****p<0.0001.

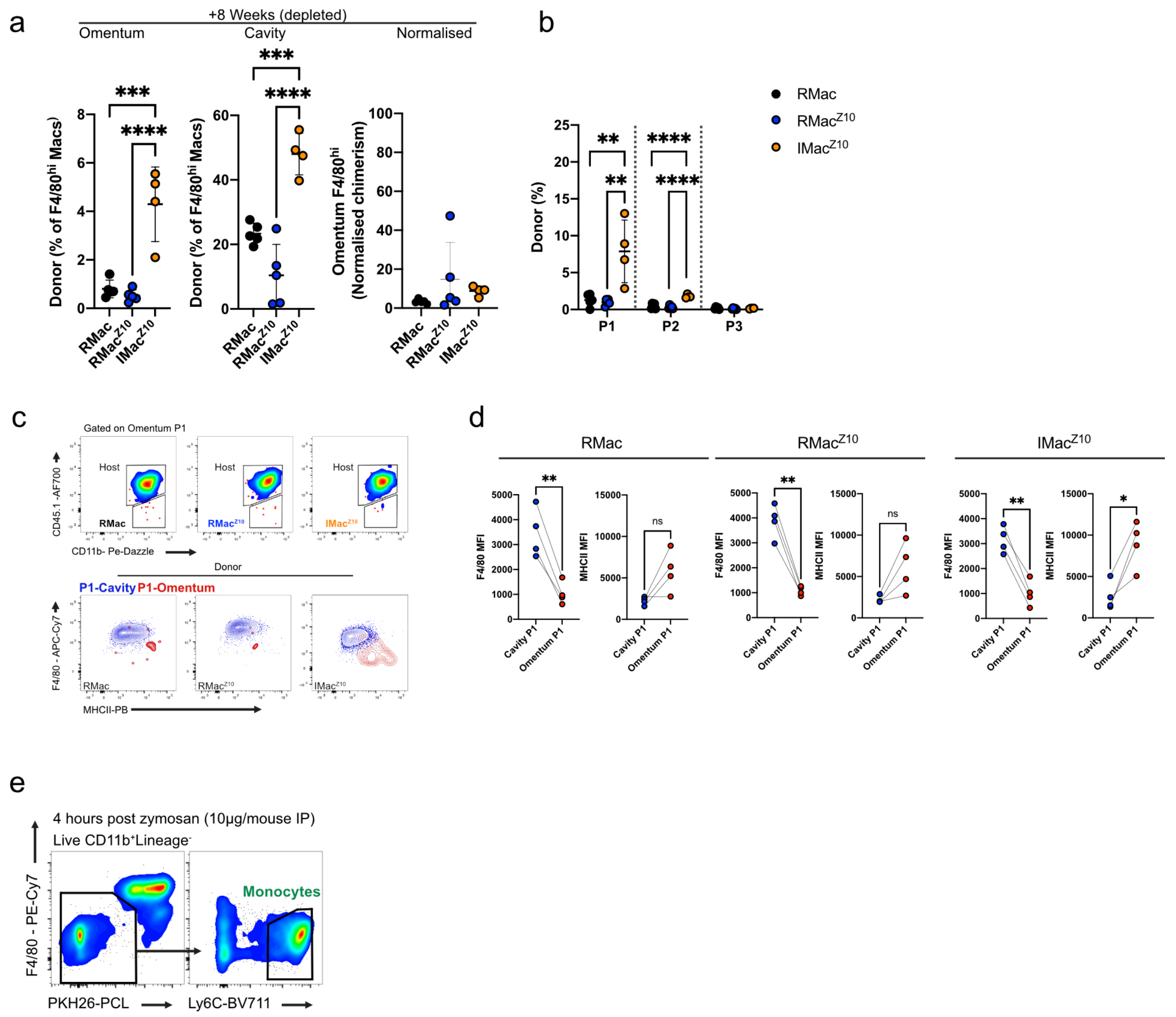

Supplementary Figure 3. Peritoneal cavity macrophage subsets migrate to macrophage-deplete omentum.

(a) Proportion of F4/80^{hi} macrophages in the omentum and cavity, and the former normalised to the latter, that are of donor origin 8 weeks post transfer into the cavity of clodronate depleted recipient mice (RMac, black, n=5; RMac^{Z10}, blue, n=5; IMac^{Z10}, orange, n=4). Statistical significance determined using one-way ANOVA with Tukey's multiple comparisons test. **(b)** Proportion of omental P1, P2 or P3 cells that are of donor origin in samples shown in (a). Statistical significance determined using two-way ANOVA with Sidak multiple comparisons adjustment.

(c) Representative identification of donor cell populations 8 weeks post transfer and representative expression of F4/80 and MHCII on donor peritoneal and omental P1 cells after transfer of the indicated populations.

(d) Quantification of F4/80 MFI and MHCII MFI on donor cells in the cavity P1 compartment and the omental P1 compartment (n=4/group). One sample from both the RMac and RMac^{Z10} group was excluded as too few cells were present to quantify MFI.

(e) Representative gating strategy to identify $Ly6C^+$ monocytes 4 hours post zymosan injection. This plot was sourced from a previously published dataset[19].

Data presented was from a single experiment, and is presented as mean ± standard deviation with symbols representing individual animals. *p* values are reported as *p<0.05, **p<0.01, ***p<0.001, ****p<0.0001.

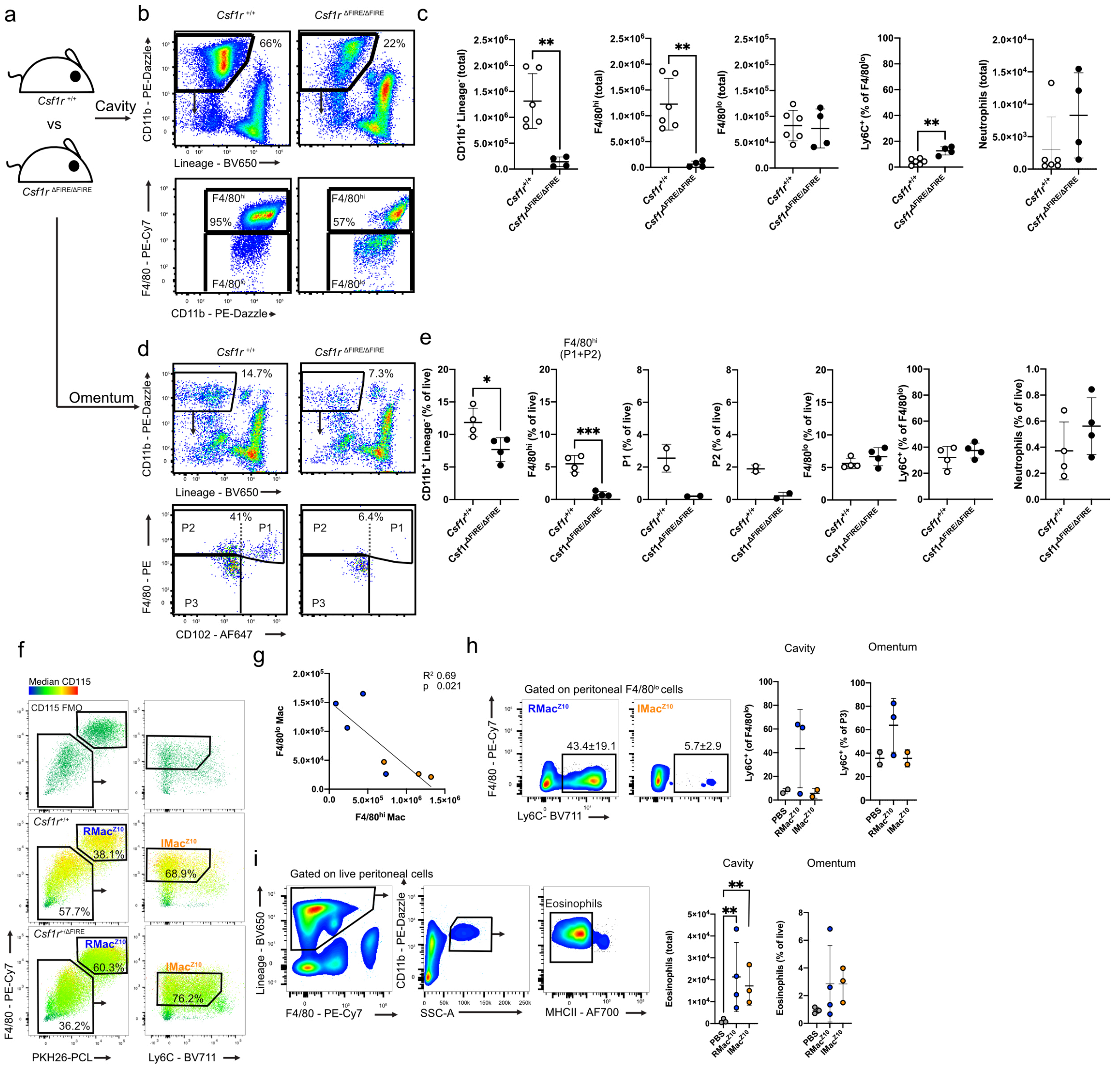

Supplementary Figure 4. Resident and inflammatory macrophages differ in capacity to repopulate peritoneal and omental niches.

(**a**) Experimental schematic.

(b) Gating strategy to identify F4/80^{hi} and F4/80^{lo} myeloid cells in the peritoneal cavity in *Csf1r*$^{+/+}$ and *Csf1r*$^{\Delta FIRE/\Delta FIRE}$ mice.

(c) Total neutrophils, CD11b^{+} Lineage^{-} myeloid cells, F4/80^{hi} and F4/80^{lo} cells and the proportion of the latter that are Ly6C^{+} monocytes in cavity in *Csf1r*$^{+/+}$ (n=4) and *Csf1r*$^{\Delta FIRE/\Delta FIRE}$ (n=3) mice. Statistical significance was determined using student's t test.

(d) Gating strategy to identify omental CD102^{+}/F4/80^{hi} macrophages (P1+P2) and CD102 F4/80^{lo} myeloid cells (P3) in *Csf1r*$^{+/+}$ and *Csf1r*$^{\Delta FIRE/\Delta FIRE}$ mice.

(e) Proportion of live omental cells that are neutrophils, CD11b^{+}Lineage^{-} myeloid cells,

F4/80^{hi} (P1+P2), CD102^{+} (P1), or CD102^{-} (P2) macrophages, or CD102^{-}F4/80^{lo} myeloid cells and the proportion of CD102^{-}F4/80^{lo} myeloid cells that are Ly6C^{+} monocytes in *Csf1r*$^{+/+}$ (n=4) and *Csf1r*$^{\Delta FIRE/\Delta FIRE}$ (n=4) mice. Statistical significance determined using student's t test.

(f) Representative gating strategy to identify RMac^{Z10} and IMac^{Z10} in *Csf1r*$^{+/+}$ and *Csf1r*$^{\Delta FIRE/\Delta FIRE}$ mice 3 days following zymosan administration.

(g) Correlation between total cavity F4/80^{lo} cells (y axis) and F4/80^{hi} cells (x axis) in *Csf1r*$^{\Delta FIRE/\Delta FIRE}$ recipients following transfer of RMac^{Z10} (blue) or IMac^{Z10} (orange). Correlation was assessed using a simple linear regression.

(h) Representative gating of Ly6C^{+} cells within the Lineage^{-}CD11b^{+}F4/80^{lo} peritoneal macrophage compartment and quantification of the absolute number of Ly6C^{+} monocytes in the cavity, and proportion of omental F4/80^{lo} myeloid cells (P3) that are Ly6C^{+}. Numbers on flow cytograms indicate mean and standard error of 3 (RMac^{Z10}) or 2 (IMac^{Z10}) mice per group.

(i) Representative gating strategy (left) and quantification (right) of peritoneal and omental eosinophils in *Csf1r*$^{\Delta FIRE/\Delta FIRE}$ recipients following transfer of RMac^{Z10} (blue) or IMac^{Z10} (orange). Statistical significance determined using one-way ANOVA with Tukey's multiple comparisons test and student's t test.

Data presented was from at least 3 experiments, and is presented as mean ± standard deviation with symbols representing individual animals. *p* values are reported as *p<0.05, **p<0.01, ***p<0.001, ****p<0.0001.

Antibody	Clone	Source	fluorochrome	Catalogue#	Dilution
CD102	3C4	Biolegend	FITC	105606	1:400
	3C4	Biolegend	Biotin	105604	1:400
CD11b	M1/70	Biolegend	Pe-Dazzle	101256	1:400
CD11c	N418	Biolegend	APC-Cy7	117324	1:200
CD16/32	2.4G2	Biolegend	Purified	101320	1:200
CD19	6D5	Biolegend	Biotin	115504	1:200
	6D5		PB	115523	1:200
CD3	17A2	Biolegend	Biotin	100244	1:200
	17A2	Biolegend	PB	100214	1:200
CD45.1	A20	Biolegend	FITC	110706	1:200
	A20	Biolegend	AF700	110724	1:200
CD45.2	104	Biolegend	AF700	109822	1:200
CD64	x54-5/7.1	Biolegend	APC	B207411	1:100
CSF1R	AFS98	Biolegend	APC	135510	1:200
F4/80	BM8	Biolegend	Pe-Cy7	123114	1:200
		Biolegend	APC-Cy7		
Ly6c	HK1.4	Biolegend	BV711	128037	1:400
Ly6g	1A8	Biolegend	Biotin	127604	1:200
	1A8	Biolegend	PB	127612	1:200
MHCII	M5/114.15.2	Biolegend	AF700	107622	1:200
	M5/114.15.2	Biolegend	BV510	107635	1:200
Siglec F	ES22-10D8	Miltenyi	Biotin	130-101-861	1:100
	E50-2440	BD	BV421	562681	1:200
Streptavidin		Biolegend	BV650	405232	1:1000
TIM4	RMT4-54	Biolegend	PE	130006	1:400
	RMT4-54	Biolegend	Pe-Cy7	130010	1:400

Supplementary Table 1: List of antibodies used for flow cytometry in this study

Antibody	Clone	Source	fluorochrome	Catalogue#	Dilution
GATA6	D61E4	Cell signaling	Purified	5851S	1:400
F4/80	CL: A3-1	Serotec	Purified	MCA497R	1:50
TIM4	RMT4-54	Biolegend	AF647	130007	1in200
α-rabbit		Life tech	AF555	A31572	
α-rat		Life tech	AF647	A21247	

Supplementary Table 2: List of antibodies used for confocal microscopy in this study
